# Crimean Congo hemorrhagic fever serosurvey in humans for identifying high-risk populations and high-risk areas in the endemic state of Gujarat, India

**DOI:** 10.1186/s12879-019-3740-x

**Published:** 2019-02-01

**Authors:** Devendra T. Mourya, Pragya D. Yadav, Yogesh K. Gurav, Prachi G. Pardeshi, Anita M. Shete, Rajlaxmi Jain, Dinkar D. Raval, Kamlesh J. Upadhyay, Deepak Y. Patil

**Affiliations:** 1 ICMR-National Institute of Virology, 20-A, Dr. Ambedkar Road, Pune, Maharashtra Pin 411001 India; 2 State Health Society, Health and Family Welfare Department, Gandhinagar, Gujarat India; 3 Department of Medicine B. J. Medical College, Ahmedabad, Gujarat India

**Keywords:** Crimean Congo hemorrhagic fever, Gujarat, Human, Serosurvey, ELISA, Ticks, Risk factors

## Abstract

**Background:**

Crimean Congo Hemorrhagic Fever (CCHF) is a highly infectious zoonotic disease of humans transmitted by *Hyalomma* ticks. Earlier studies have shown CCHF seroprevalence in livestock throughout India, yet sporadic outbreaks have been recorded mostly from the Gujarat state of India since 2011. Occupational vulnerability to CCHF for animal handlers, veterinarians, abattoir workers, and healthcare workers has been documented. The current study was planned to determine the seroprevalence of CCHF with an intention to identify the high -risk population and high -risk areas from Gujarat state, India.

**Methods:**

Based on the socio-clinical data, the human population of Gujarat was divided into eight categories viz. A: CCHF affected person/house/close contact, B: Neighborhood contacts, C: Animal handlers, D: General population, E: Farmers, F: Abattoir workers, G: Veterinarian, H: Healthcare workers. A total of 4978 human serum samples were collected from 33 districts of Gujarat during year 2015, 2016 and 2017. All the samples were screened for the presence of anti-CCHFV IgG using indigenously developed anti-CCHFV IgG ELISA. Univariate regression analysis was performed to recognize significant risk factors for CCHF seropositivity.

**Results:**

Twenty-five serum samples were found to be positive with an overall CCHF human seropositivity of 0.5% (95% CI 0.30–0.74%). Gender predisposition to CCHF prevalence was observed in males (OR: 2.80; *p-value*: 0.020). The risk for seropositivity increased sevenfold when a person was in contact or neighbor with a CCHF case (OR 7.02; *p-value:* < 0.0001). No significant difference in seropositivity was observed within different age groups. Veterinarians, healthcare workers, and control group were found to be seronegative for CCHF.

**Conclusions:**

In-spite of CCHF sporadic outbreaks reported in Gujarat, the seropositivity for CCHF in the state was low as compared to other endemic countries. Males, close contacts and neighbors were identified as a high-risk population for CCHF infection. To recognize the high-risk area, tick screening and animal serosurvey would be a wiser choice. The study also suggests circulation and under diagnoses of CCHFV in the naïve regions of Gujarat.

## Background

Crimean Congo hemorrhagic fever (CCHF) is a tick-borne zoonotic disease caused by CCHF virus (CCHFV) belonging to family *Nairoviridae,* genus *Orthonairovirus* [1]. This virus is known to cause case fatality rate of up to 80% in humans [2, 3]. The disease is prevalent in Africa, Asia, Southeast Europe and the Middle East [3, 4]. CCHFV infection is highly infectious with a high rate of human-to-human transmission. Domestic animals and *Hyalomma* ticks play an essential role in the amplification of virus and transmission to human. Nosocomial infection, bite of infected ticks, crushing ticks with bare hands and contact with the blood of infected animals/ humans tissue fluids are the major routes of transmission of CCHF to humans. Available information suggests that CCHF cases mainly occur as a result of occupational exposure among abattoir workers, farmers, veterinarians, and healthcare workers [5].

India reported its first CCHF case in the year 2011 from Ahmedabad, Gujarat [6]. Since then, several sporadic cases and outbreaks of CCHF have been reported mostly from Gujarat and few from Rajasthan and Uttar Pradesh States of India [7, 8]. Over some time, the majority of CCHF cases have been published from various districts of Gujarat thus making Gujarat an endemic state for CCHF disease in India. Though serological evidence against CCHF in humans has been reported in India [9, 10], a systematic data on CCHF seroprevalence is lacking. CCHF IgG seropositivity of 5.4% in cattle and 10.99% in sheep and goats from most of the states of India has been recorded earlier without any remarkable difference between Gujarat and the other states thus indicating the prevalence of this virus throughout the country [11]. India has well-organized animal husbandry, and a larger rural population depends for their livelihood by maintaining livestock. Despite the countrywide presence of the vector species and domestic reservoir animals, CCHF human cases and human outbreaks have been reported predominantly only from one state in India, i.e., Gujarat. Intending to understand CCHF seroprevalence and to identify high-risk populations and high-risk areas in Gujarat, the present cross-sectional serosurvey was undertaken in the human community.

## Methods

### Study design

#### The study area, period and characteristic of study population

All the 33 districts of Gujarat were considered as a site for sample collection from the human population during the year 2015, 2016 and 2017. Human serum samples were collected from Ahmedabad, Amreli, Patan, Aravalli, Kheda, Morbi, Kutch, Surendranagar, Mahesana, Jamnagar, Botad, Valsad, Anand, Rajkot, Panchmahal, Devbhoomi Dwarka, Banaskantha, Bharuch, Bhavnagar, Dahod, Gir-Somnath, Junagadh, Mahisagar, Narmada, Navsari, Sabarakantha, Surat, Tapi, Vadodara, Chhota Udepur, Porbandar, Gandhinagar and Dang districts of Gujarat State.

The transmission of CCHF infection to humans through contact with CCHFV infected individuals, animals and bite of ticks is well known. Since 2011, many sporadic CCHF cases were reported from Gujarat State making it an endemic state for CCHF [7, 9]. Based on available record history, individual CCHF survivors were identified, and their households were traced. The categories were designed based on socio-clinical data of the subject and the occupational risk to CCHF infection. Study population belonging to Gujarat State was categorized as **Category A:** CCHF affected person/CCHF affected house/caretaker of the patient, close contacts, person involved in transportation of CCHF cases to hospital; **Category B:** All the family members (except children aged ≤5 yrs.) of neighborhood (i.e. within 50–100 m of the radius of confirmed CCHF case house; **Category C:** Animal handlers (person living in close contact with livestock, persons grazing the livestock) **Category D:** General population (low-risk group); **Category E:** Farmers; **Category F:** Abattoir Worker; **Category G:** Veterinarians; **Category H:** Healthcare workers. Samples of pregnant women and critically ill patients were not collected. Stratified purposive sampling was planned from Category A and B category, and random sampling was done from categories C, D, E, F, G and H..

#### Sample collection and processing

The Institutional Human Ethics Committee of ICMR-NIV, Pune (IHEC No, NIV/IEC/2017/37/42) approved the study. Informed written consent was obtained from all the subjects and parent/guardian in case of a child (< 18 yrs) subjects from the study population. Blood sample (3–5 mL) was collected from the study population in clot activator tube and transported to ICMR-NIV, Pune in a cold chain. After the receipt of the samples at NIV, each sample was checked for its quality and condition. Serum was separated from the blood sample of each subject and further aliquoted as 50 μL, 250 μL, stock and stored at − 20 °C until further use. Each sample was registered in laboratory information management system software by giving a unique identification number/anonymous code. A demographic and socio-clinical detail of every subject was also recorded. The complete process of sampling from the transportation of specimen to the laboratory investigations was done with utmost precaution, which includes; collection of samples by trained personnel using personal protective equipment (apron, mask, and gloves) in a leak-proof container and transportation by maintaining cold chain conditions. Aliquoting and testing of samples were carried out in a BSL-2 facility.

#### Development of indigenous anti-CCHF human IgG ELISA, its validation and screening of human serum samples

The human serum samples were tested for the presence of anti-CCHFV IgG antibodies using in-house developed CCHF Human IgG ELISA as defined below. Micro-well plates were coated with Gamma-inactivated CCHFV infected Vero CCL81 cell lysate antigen strain number NIV11704 (Rows A-D) and uninfected Vero CCL81 cell lysate (Rows E-H) at 1:20 dilution in carbonate buffer (pH 9.2, 0.025 M) and kept overnight at 4 °C (100 μL/well). The plate was blocked with liquid plate sealer (LPS) (Candor Biosciences, Germany) (100 μL/well) and kept at room temperature for 2 h. Further, these blocked and dried plates were stored at 4 °C. Serum sample (diluted 1:600 in 1% BSA in 10 mM PBS containing 0.1% Tween) was added (100 μL/well) in respective positive as well as negative antigen-coated wells and incubated for 1 h at 37 °C. Subsequently, anti-human IgG HRP (1,30,000, 100 μL/well) (Pierce) was added to the wells and incubated for 1 h at 37 °C. After the incubation, TMB (3,3′, 5,5’-Tetramethylbenzidine) substrate (100 μL/well) was added to the wells and for 10 min incubated at room temperature. Finally, the reaction was terminated using 1 N H_2_SO_4_ and absorbance was measured at 450 nm. At the end of each step of this assay, all the wells were washed five times using ten mM PBS, pH 6.8, and 0.1% Tween-20 (Sigma, St. Louis, MO, USA). Appropriate controls were included in each test. For an unknown sample (test sample), when OD value of the sample with CCHF antigen was more than 0.2 and the P/N ratio more than 1.5, the sample considered as “Positive.” Less than 1.5 P/N ratio was considered as “Negative” for the presence of anti-CCHFV IgG antibodies.

For validation of the assay, 134 sera samples (5 CCHF IgG antibody positive and 129 CCHF IgG antibody negative samples) tested by Vector Best kit, Russia, were compared with NIV anti-CCHF Human IgG ELISA kit. The assay was also provided for external validation to three national level laboratories. Cross reactivity of the assay was checked with Ganjam virus of Family *Nairoviridae,* Rift valley fever virus of *Phenuiviridae,* Dengue virus, Kyasanur Forest Disease virus and Japanese Encephalitis virus of *Flaviviridae*, Chikungunya virus of *Togaviridae* and Hanta virus of *Hantaviridae.* A total of 4978 human serum samples were screened using this indigenous ELISA for the presence of anti-CCHFV IgG antibodies.

#### Data analysis of results of samples screened using anti-CCHF human IgG ELISA

To identify the risk factors, the result obtained by testing 4978 human serum samples by indigenous anti-CCHF Human IgG ELISA was analyzed by univariate regression analysis using Epi-info 7 (7.2.1.0, CDC, Atlanta, GA, USA) and MedCalc Software bvba, version 18 (1993–2018). For plotting state of Gujarat map, the svg file was downloaded from https://commons.wikimedia.org/wiki/File:India_Gujarat_location_map.svg and edited as required.

## Results

### The study area, period and characteristic of study population

Sample collection was done from 91 villages of 33 districts of Gujarat throughout 3 years (2015, 2016 and 2017). The number of samples collected from each district of Gujarat State is given in Table 1. The study included subjects ranging from 5 to 90 years of age with maximum collection from age group, 20 - < 40 yrs. (*n* = 2126), while the minimum collection was done from age group 80 - < 100 yrs. (*n* = 14). A total of 2587 female subjects (52%) and 2391 male subjects (48%) were involved in this study. Category-wise male and female sample distribution is given in Table 2.

**Table 1:** District-wise distribution of human serum samples collected from Gujarat state

District- wise human samples collected from Gujarat State				
Sr. No.	District	Total samples collected	Percent	95% CI
1	Ahmedabad	100	2.01%	1.65–2.44
2	Amreli	196	3.94%	3.43–4.51
3	Anand	100	2.01%	1.65–2.44
4	Aravalli	89	1.79%	1.46–2.19
5	Kheda	96	1.93%	1.58–2.35
6	Kutch	100	2.01%	1.65–2.44
7	Botad	100	2.01%	1.65–2.44
8	Morbi	100	2.01%	1.65–2.44
9	Rajkot	100	2.01%	1.65–2.44
10	Valsad	200	4.02%	3.51–4.60
11	Jamnagar	90	1.81%	1.47–2.22
12	Patan	100	2.01%	1.65–2.44
13	Mehsana	77	1.55%	1.24–1.93
14	Surendranagar	100	2.01%	1.65–2.44
15	Panchmahal	200	4.02%	3.51–4.60
16	Devbhoomi Dwarka	201	4.04%	3.53–4.62
17	Porbandar	103	2.07%	1.71–2.50
18	Banaskantha	191	3.84%	3.34–4.41
19	Girsomnath	200	4.02%	3.51–4.60
20	Gandhinagar	200	4.02%	3.51–4.60
21	Narmada	197	3.96%	3.45–4.54
22	Navsari	206	4.14%	3.62–4.73
23	Junagadh	200	4.02%	3.51–4.60
24	Mahisagar	182	3.66%	3.17–4.21
25	Sabarkantha	203	4.08%	3.56–4.66
26	Surat	197	3.96%	3.45–4.54
27	Tapi	200	4.02%	3.51–4.60
28	Vadodara	200	4.02%	3.51–4.60
29	Bharuch	190	3.82%	3.32–4.39
30	Bhavnagar	114	2.29%	1.91–2.74
31	Chhota Udepur	195	3.92%	3.41–4.49
32	Dahod	200	4.02%	3.51–4.60
33	Dang	51	1.02%	0.78–1.34
	Total samples collected	4978	100.00%

**Table 2:** Category-wise (A-H) distribution of total human serum samples from the male and female population

Subject category	Total number of serum samples (%)	Number of female serum samples (%)	Number of male serum samples (%)
A: CCHF Affected Person/House/Close contact	199 (3.99)	98 (49.24%)	101 (50.75%)
B: Neighborhood contacts	829 (16.65)	487 (58.74%)	342 (41.25%)
C: Animal Handler	723 (14.52)	404 (55.87%)	319 (44.12%)
D: General population	1677 (33.68)	963 (57.42%)	714 (42.57%)
E: Farmer	1035 (20.79)	440 (42.51%)	595 (57.48%)
F: Abattoir worker	104 (2.08)	32 (30.76%)	72 (69.23%)
G: Veterinary worker	104 (2.08)	4 (3.84%)	100 (96.15%)
H: Healthcare worker	307 (6.16)	159 (51.79%)	148 (48.20%)
Total samples	4978	2587	2391

### Development of indigenous anti-CCHF human IgG ELISA, its validation and screening of human serum samples

The sensitivity and specificity for this assay were observed to be 85.70 and 98.44%, respectively using vector Best, Russia assay as the gold standard (Table 3). The external national level laboratories validated the assay in 100% concordance. The O.D range was observed to be 0.250–1.610 for positive samples and 0.010–0.159 for negative samples. Twenty five human serum samples were found to be positive by ELISA out of 4978 samples screened. No cross-reactivity was observed with Ganjam virus, Rift valley fever virus, Dengue virus, Kyasanur Forest Disease virus, Japanese Encephalitis virus, Chikungunya virus, and Hanta virus. Thus, the assay was found to be specific*.*

**Table 3:** Comparison of Sensitivity and Specificity of Anti-CCHF Human IgG ELISA assay (NIV kit) versus Gold standard test (Vector Best Kit, Russia)

Anti-CCHF IgG positive and negative samples tested by Vector Best kit, Russia	Total number of samples tested by both the kits	Anti-CCHF Human IgG ELISA (NIV kit)	
		IgG antibody Positive	IgG antibody Negative
CCHF IgG Positive	7	6	1
CCHF IgG Negative	129	2	127
Total	136	8	128

Anti-CCHF Human IgG ELISA (NIV kit) Sensitivity =85.7%

Anti-CCHF Human IgG ELISA (NIV kit) Specificity =98.44%

### Data analysis of human serum samples screened using anti-CCHF human IgG ELISA

**Fig. 1:**
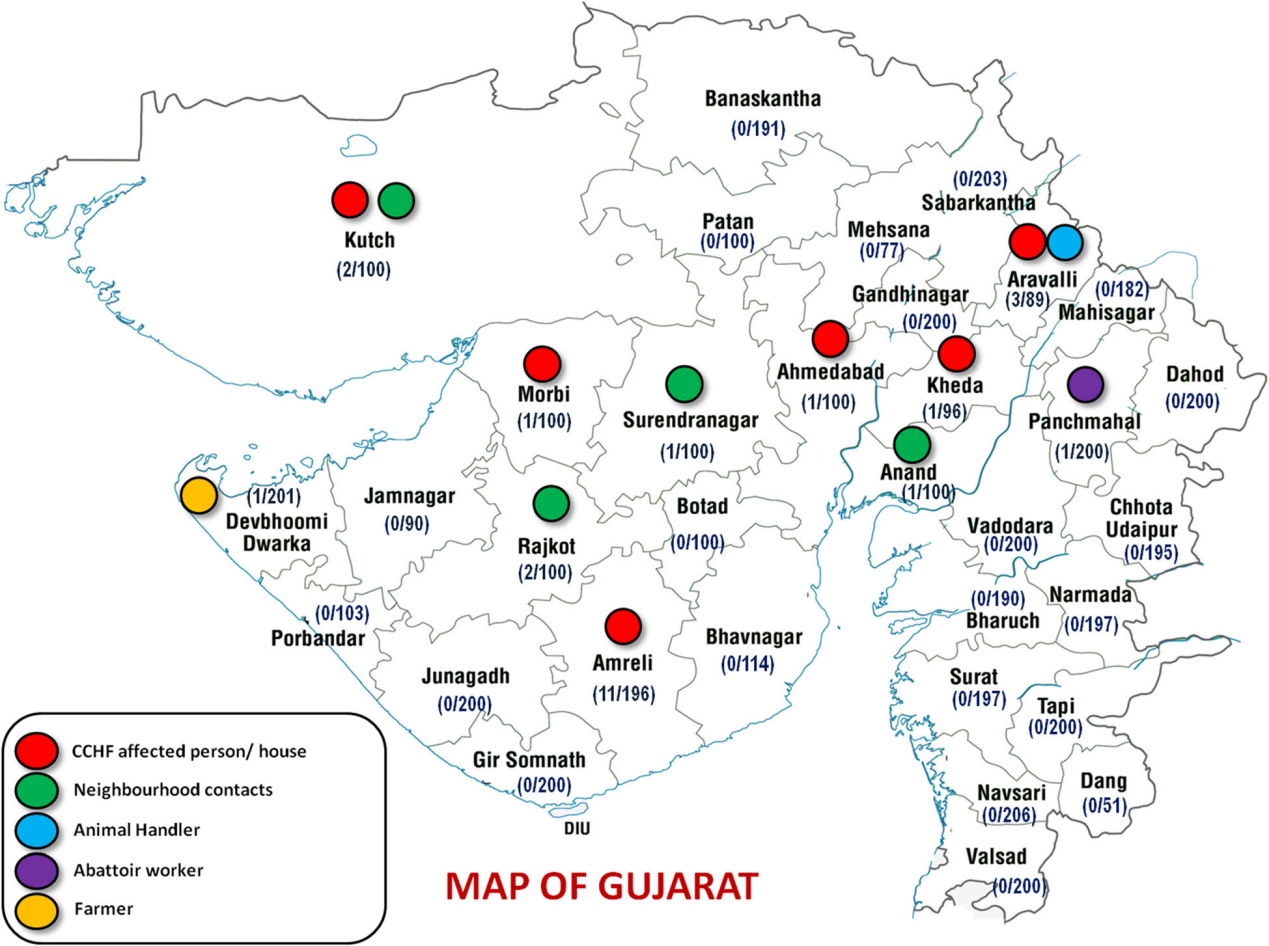
CCHF seropositivity in different districts of Gujarat state based on subject categories. The numbers in bracket indicatethe a total number of positive samples/total number of tested samples from the district. (For plotting state of Gujarat map, the svg file was downloaded from https://commons.wikimedia.org/wiki/File:India_Gujarat_location_map.svg and then edited as per requirement)

Overall anti-CCHFV Human IgG seroprevalence was found to be 0.5% (95%CI 0.3%-0. 74%) in the state of Gujarat, India based on 4978 human serum samples screened. Out of 25 positive samples, 17 samples belonged to the category “A” (CCHF affected cases/house/close contacts) from various districts: Amreli (*n* = 11), Aravalli (*n* = 2), Kheda (*n* = 1), Morbi (*n* = 1), Kutch (*n* = 1) and Ahmedabad (*n* = 1) districts, five samples belonged to category “B” (Neighborhood) from Rajkot (*n* = 2), Kutch (*n* = 1), Anand (*n* = 1) and Surendranagar (*n* = 1) district. One sample belonged to category “C” (Animal Handlers) from Aravalli district, one sample was from Category “E” (Famer) and one from Category “F” (Abattoir worker) belonging to districts Devbhoomi Dwarka and Panchmahal respectively. No positivity was recorded from subjects belonging Category “G” (Veterinary workers), Category “H” (healthcare workers) and low-risk group, i.e., Category “D” (general population) (Fig. 1).

To find high-risk factors, seropositivity for parameters like the gender, age group, subject category and district of the subject (Table 4) were analyzed. Categories to which the subject belonged, the gender of the subject and District was found to be the risk factors to CCHF infection. Maximum positivity was observed from “Category A” (OR-88.33; *p-value*: < 0.0001) when compared to other categories, which was due to the purposive sampling of known CCHF survivors from the state. Amreli district recorded highest IgG positivity (11/196) making it a high-risk area of Gujarat (OR 11.28; *p-*value*:* 0.020). We also found that the risk for seropositivity increased sevenfold when a person was in contact or neighbor with a CCHF case (OR 7.02; *p-*value*:* < 0.0001). The seropositivity of 1.49% [95% CI 0.99–2.22%) was seen from CCHF affected districts [districts with recorded CCHF case]. Only two positive samples were confirmed from CCHF unaffected districts [districts with no recorded CCHF case] [0.06% (95% CI 0.02–0.21%)] belonging to Devbhoomi Dwarka and Pachmahal districts.

**Table 4:** Univariate regression analysis of CCHF seropositive data generated from subjects belonging to all the districts of Gujarat State

Risk factors	Negative	Positive (Percent %)	Total	Odds ratio	95% CI	*p-*value
Gender						
Male	2373	18 (0.75)	2391	2.8033	1.16–6.72	0.0209^*^
Female	2580	7 (0.27)	2587	1		
Age groups						
0 - < 20	498	1 (0.20)	499	1		
20 - < 40	2126	7 (0.33)	2133	1.6343	0.20–13.31	0.6462
40 - < 60	1773	14 (0.78)	1787	3.9015	0.51–29.74	0.189
60 - < 80	542	3 (0.55)	545	2.7565	0.28–26.58	0.3806
80 - < 100	14	0	14	0		
Subject Category						
A: CCHF affected person/ house/ close contacts	182	17 (8.54%)	199	88.3317	11.68–667.55	< 0.0001^*^
F: Abattoir workers	103	1 (0.96%)	104	9.9423	0.61–160.12	0.1053
B: Neighborhood	824	5 (0.60%)	829	6.2364	0.72–53.48	0.095
C: Animal grazers	722	1 (0.14%)	723	1.4302	0.08–22.93	0.8004
E: Farmers	1034	1 (0.10%)	1035	1		
D: General population	1677	0	1677	0		
G: Veterinarian	104	0	104	0		
H: Healthcare personnel	307	0	307	0		
Seropositive Districts						
Ahmedabad	99	1 (1.00%)	100	2.01	0.12–32.47	0.6228
Amreli	185	11 (5.61%)	196	11.2806	1.44–88.20	0.0209^*^
Aravalli	86	3 (3.37%)	89	6.7753	0.69–66.03	0.0996
Rajkot	98	2 (2.00%)	100	4.02	0.36–44.86	0.2583
Kutch	98	2 (2.00%)	100	4.02	0.36–44.86	0.2583
Surendranagar	99	1 (1.00%)	100	2.01	0.12–32.47	0.6228
Anand	99	1 (1.00%)	100	2.01	0.12–32.47	0.6228
Kheda	95	1 (1.04%)	96	2.0937	0.12–33.83	0.6027
Morbi	99	1 (1.00%)	100	2.01	0.12–32.47	0.6228
Panchmahal	199	1 (0.50%)	200	1.005	0.06–16.17	0.9972
Devbhoomi Dwarka	200	1 (0.50%)	201	1		

^*^ indicate significant values

When the CCHF positive subjects history was traced back, it was found that, out of 17 positive subjects of “Category A” (17/199), six were identified as primary cases (all males) with a history of tick bite or animal contact; seven were close contacts (both males and females) while four had unknown history of illness. Positivity was observed in both males and females from Neighborhood contacts (5/829), while only men were seropositive in case of animal handlers (1/723), farmers (1/1035) and abattoir workers (1/104). Therefore, in this study, gender vulnerability of males to CCHF infection was found to be significant as compared to females (OR: 2.80; *p-*value: 0.020). Due to unavailability of tick-bite history for all the subjects and livestock contact for subjects belonging to Category A and B, these factors could not be analyzed and were not included in the present study. Other parameters like age of the subject did not contribute as a risk factor for CCHF infection (Table 4).

## Discussion

The present study was designed to recognize the circulation of CCHFV in the endemic state of Gujarat with an intention to identify high-risk population and high-risk areas of the state. This study was intended to evaluate a vast number of subjects (4978) throughout 3 years. In general, CCHF human seropositivity in the Gujarat state was 0.5% (95%CI 0.3%–0.74%), which was deciphered to be reasonably low as against the seropositivity recorded in other endemic countries of the world like Sistan and Baluchistan province of Pakistan (68%), Iraq (12%), Turkey (12.8%), Greece (4.9%), Kosovo (4%) and Bulgaria (3.7%), [12–17]. CCHF IgG prevalence from healthy family members and contacts of CCHF patients (Category “A”) in Gujarat was found to be 8.53% (11/199) as against 36% recorded in southwestern Mauritania [18], whereas, veterinarians (0/104), healthcare workers (0/307) and control group, i.e., General population (0/1677) included in the study was found to be seronegative. CCHF affected districts had more seropositivity in comparison to CCHF unaffected districts, overall suggesting a meagre human-human transmission rate of the Indian CCHFV as compared to other CCHF viruses circulating in the world. The restricted spread of this virus in India could also have been due to timely diagnosis, control strategies and active surveillance since the first reported CCHF case in 2011 from Gujarat. This study was successful in identifying the gender predisposition to CCHFV. However, there were few limitations like unavailability of tick-bite data for all the subjects and livestock contact data for all the subjects belonging to A and B categories. To obtain exposure data, we tracked the history record of 25 CCHF seropositive subjects. It was found that, out of 25 subjects, six were index cases and all were males with a history of either contact with animals or tick-bite. Indian rural population is economically dependent on animal husbandry practices and various farming activities. Mostly, in India, males dominate the agricultural occupational activities. Hence, gender susceptibility of the men for CCHF infection was observed. In nature, domestic animals play an essential role as reservoirs for CCHF virus. Once they receive an infected tick bite, they develop transient viremia of short duration, and during that period other ticks attached to the body of the animal get infected with this virus [19]. Earlier studies have shown a very few vector ticks were found affected when collected from the cattle sheds of index cases households [20], suggesting that probably all the ticks don’t get infected. In this scenario, *Hyalomma* tick being a three-host vector tick, larval and nymphal stages, mainly play a role in the further dissemination of this infection to other animals and humans. The human index cases are mostly due to accidental bites of infected ticks as they reside in the very close proximity of livestock. In such situations, determining major risk areas for human infection is difficult due to close human-animals interphase in every household in all rural areas. This also requires necessary information on the presence of the high prevalence of this infection in domestic animals. Out of the 33 districts, highest seropositivity was observed from Amreli district (11/196), and therefore that district, in particular, was identified as a high-risk area for CCHF infection, however, all the 11 positive subjects from Amreli were CCHF survivors as suggested by ICMR-NIV data and the outbreak investigation carried out from Amreli during the 2013 CCHF outbreak [7] giving a biased result. None of the other districts of Gujarat were found to be a high-risk area. Earlier data of the livestock CCHF serosurvey suggest seroprevalence throughout India [11].

Conversely, a large number of human outbreaks continue to be reported only from the state of Gujarat. To identify high-risk areas, screening of ticks or nymphs for the presence of CCHFV and livestock CCHF serosurvey could be a wiser choice than human serosurvey. Seropositivity from relatively naïve districts of Gujarat suggests under-diagnosed CCHF in Gujarat.

## Conclusions

CCHF serosurvey in humans from the endemic state of Gujarat reported a low CCHF seropositivity for CCHF as against other endemic regions of the world. Data suggests gender specificity towards CCHF infection in India due to occupational exposure to animals and tick-bite. Amreli District was identified as a high-risk area as all the positive subjects were CCHF survivors, and none of the other districts were identified as a high-risk area. Seropositivity in comparatively naïve areas like Panchmahal and Devbhoomi Dwarka suggests under-diagnosed CCHF disease in such areas. Assessment of CCHF prevalence in livestock and screening of ticks and nymphs for the presence of CCHFV would give a better understanding for identifying the CCHF high-risk areas.

## Abbreviations

CCHF: Crimean Congo hemorrhagic fever
CCHFV: Crimean Congo hemorrhagic fever virus
IgG: Immunoglobin G
ICMR-NIV: Indian Council of Medical Research-National Institute of Virology
RT-PCR: Reverse transcriptase polymerase chain reaction

## References

[1] Adams MJ, Lefkowitz EJ, King AMQ, Harrach B, Harrison RL (2017). Changes to taxonomy and the international code of virus classification and nomenclature ratified by the international committee on taxonomy of viruses (2017). Arch Virol.

[2] Whitehouse CA (2004). Crimean-Congo hemorrhagic fever. Antivir Res.

[3] Ergönül O (2006). Crimean-Congo haemorrhagic fever. Lancet Infect Dis.

[4] Hoogstraal H (1979). Review article 1: the epidemiology of tick-borne Crimean-Congo hemorrhagic fever in Asia, Europe, and Africa. J Med Entomol.

[5] Centers for Disease Control (CDC) (1988). Management of patients with suspected viral hemorrhagic fever. MMWR Morb Mortal Wkly Rep.

[6] Mishra AC, Mehta M, Mourya DT, Gandhi S (2011). Crimean-Congo haemorrhagic fever in India. Lancet.

[7] Yadav PD, Gurav YK, Mistry M, Shete AM, Sarkale P (2014). Emergence of Crimean-Congo hemorrhagic fever in Amreli district of Gujarat state, India, June to July 2013. Int J Infect Dis.

[8] Yadav PD, Patil DY, Shete AM, Kokate P, Goyal P (2016). Nosocomial infection of CCHF among healthcare workers in Rajasthan, India. BMC Infect Dis.

[9] Gurav YK, Yadav PD, Deostwar AR, Dhruwey VS, Shete AM, Chaubal GY (2014). Serosurvey of Crimean Congo Haemorrhagic fever (CCHF) among high risk group during 2011 and 2013 CCHF outbreak in Gujarat, India. Ind J Applied Res.

[10] Shanmugam J, Smirnova SE, Chumakov MP (1976). Presence of antibody to arboviruses of the Crimean Haemorrhagic Fever-Congo (CHF-Congo) group in human beings and domestic animals in India. Indian J Med Res.

[11] Mourya DT, Yadav PD, Shete AM, Padmakar SS, Sarkale PC (2015). Cross-sectional Serosurvey of Crimean-Congo Hemorrhagic Fever Virus IgG in Livestock, India, 2013–2014. Emerg Infect Dis.

[12] Bodur H, Akinci E, Ascioglu S, Onguru P, Uyar Y (2012). Subclinical infection with Crimean-Congo hemorrhagic fever virus, Turkey. Emerg Infect Dis.

[13] Chinikar S, Ghiasi SM, Naddaf S, Piazak N, Moradi M, Razavi MR, Afzali N (2012). Fever in Humans with High-Risk Professions Livingin Enzootic Regions of Isfahan Province of Iran and Genetic Analysis of Circulating Strains. VBZ.

[14] Shahhosseini N, Jafarbekloo A, Telmadarraiy Z, Chinikar S, Haeri A, Nowotny N (2017). Co-circulation of Crimean-Congo Hemorrhagic Fever virus strains Asia 1 and two between the border of Iran and Pakistan. Heliyon.

[15] Sidira P, Maltezou HC, Haidich AB, Papa A (2012). Seroepidemiological study of Crimean-Congo hemorrhagic fever in Greece, 2009–2010. Clin Microbiol Infect.

[16] Christova I, Gladnishka T, Taseva E, Kalvatchev N, Tsergouli K (2013). Seroprevalence of Crimean-Congo hemorrhagic fever virus, Bulgaria. Emerg Infect Dis.

[17] Christova I, Panayotova E, Trifonova I, Taseva E, Hristova T, et al. Country-wide seroprevalence studies on Crimean-Congo hemorrhagic fever and hantavirus infections in general population of Bulgaria. J Med Virol. 2017;89:1720-25. 10.1002/jmv.24868 28561377

[18] Gonzalez P, LeGuenno B, Guillaud M, Wilson ML (1990). A fatal case of Crimean-Congo haemorrhagic fever in Mauritania: Virological and serological evidence suggesting epidemic transmission. Tras R Soc Trop Med Hyg.

[19] Swanepoel R, Paweska JT, Palmer SR, Soulsby L, Torgerson PR, Brown DWG (2011). Crimean-Congo hemorrhagic fever. Oxford Textbook of Zoonosis: Biology, Clinical Practice, and Public Health Control.

[20] Mourya DT, Yadav PD, Shete A, Majumdar TP, Kanani A, Kapadia D (2014). Serosurvey of Crimean-Congo hemorrhagic fever virus in domestic animals, Gujarat, India, 2013. Vector Borne Zoonotic Dis.

